# Ni-Doped SFM Double-Perovskite Electrocatalyst for High-Performance Symmetrical Direct-Ammonia-Fed Solid Oxide Fuel Cells

**DOI:** 10.1021/acsami.4c07968

**Published:** 2024-09-26

**Authors:** Or Rahumi, Manasa Kumar Rath, Louisa Meshi, Ilia Rozenblium, Konstantin Borodianskiy

**Affiliations:** † Department of Chemical Engineering, Ariel University, Ariel 40700, Israel; ‡ Elcogen AS, 23 Valukoja, 11415 Tallinn, Estonia; § Department of Materials Engineering, Ben-Gurion University of the Negev, Beer-Sheva 84105, Israel

**Keywords:** solid oxide fuel cell, direct-ammonia-fed cell, FeNi_3_ nanocatalyst, electrocatalysis, exsolution, direct ink writing

## Abstract

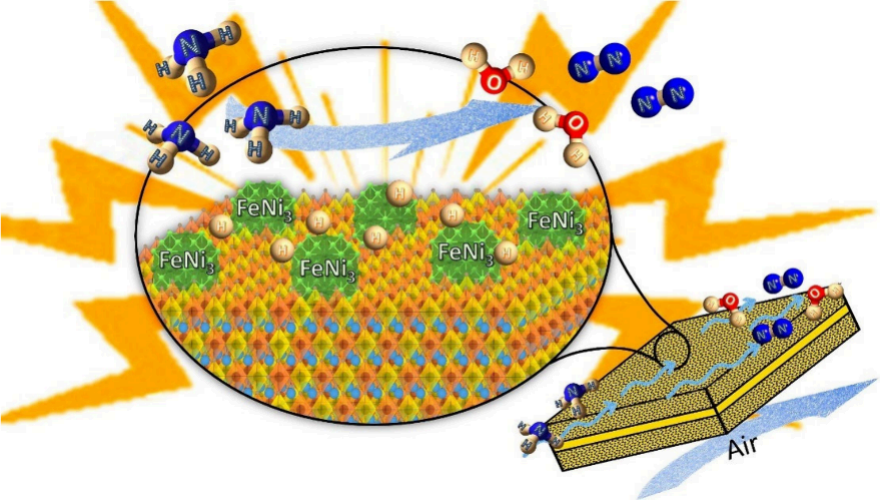

Ammonia has emerged as a promising fuel for solid oxide fuel cells (SOFCs) owing to its high energy density, high hydrogen content, and carbon-free nature. Herein, the electrocatalytic potential of a novel Ni-doped SFM double-perovskite (Sr_1.9_Fe_0.4_Ni_0.1_Mo_0.5_O_6−δ_) is studied, for the first time, as an alternative anode material for symmetrical direct-ammonia SOFCs. Scanning and transmission electron microscopy characterization has revealed the exsolution of Ni–Fe nanoparticles (NPs) from the parent Sr_2_Fe_1.5_Mo_0.5_O_6_ under anode conditions, and X-ray diffraction has identified the FeNi_3_ phase after exposure to ammonia at 800 °C. The active-exsolved NPs contribute to achieving a maximal ammonia conversion rate of 97.9% within the cell’s operating temperatures (550–800 °C). Utilizing 3D-printed symmetrical cells with SFNM-GDC electrodes, the study demonstrates comparable polarization resistances and peak power densities of 430 and 416 mW cm^–2^ for H_2_ and NH_3_ fuels, respectively, with long-term stability and a negligible voltage loss of 0.48% per 100 h during ammonia-fed extended galvanostatic operation. Finally, the ammonia consumption mechanism is elucidated as a multistep process involving ammonia decomposition, followed by hydrogen oxidation. This study provides a promising avenue for improving the performance and stability of ammonia-based SOFCs for potential applications in clean energy conversion technologies.

## Introduction

With the expansion of the global population and depletion of fossil fuel reserves, the demand for sustainable energy solutions is increasing, emphasizing the need to mitigate the negative impact of greenhouse gases. Solid oxide fuel cells (SOFCs) have gained prominence as electrochemical devices that directly convert chemical energy into electrical power,^1^ being embedded in stationary power generation systems,^2^ transportation,^3^ and building energy applications.^4^ Notably, SOFCs offer key advantages such as high efficiency,^5^ fuel flexibility,^6−8^ and robust solid-state construction, making them attractive for sustainable energy technology development.^9^

Hydrogen fuel is a promising energy storage candidate that produces clean steam upon oxidation (HOR) (Reaction R1). However, challenges related to storage, transportation, and relatively low energy density have limited their commercial use. Consequently, there has been growing interest in ammonia as a fuel because of its high energy density, hydrogen content, carbon-free composition, and well-established global distribution and storage infrastructure.^10^ A multifunctional electrode material is essential for effectively utilizing ammonia in fuel cells, exhibiting high activity for both the ammonia decomposition reaction (ADR) (Reaction R2) and the subsequent HOR at the anode.

SOFC reactions:
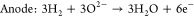
R1
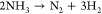
R2
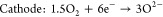
R3In the realm of ADR single-metal catalysts, the activity of Ni-based catalysts ranks second only to Ru among metal-based catalysts, including Rh, Co, Ir, Pd, and Pt.^11−13^ However, the high cost and scarcity of Ru-based catalysts limit their large-scale applications. An economically viable alternative is Fe, which is an abundant transition metal that has been widely studied as an ADR catalyst.^14,15^ Bimetallic catalysts, such as Ni–Fe^16−19^ and Fe–Mo^20^ alloys, have shown improved stability and activity compared with monometallic catalysts.

Although conventional Ni-cermet anodes fed with NH_3_ fuel have demonstrated a satisfactory performance,^21−23^ issues such as Ni-nitride formation and coarsening during prolonged cell operation, leading to anode microstructure destruction and cell failure, remain unresolved.^24,25^ Perovskite oxides have garnered significant attention as SOFC anodes owing to their chemical and physical stability and compositional and structural engineering capabilities.^26−28^ Decorating the surface of perovskite oxides with catalytically active nanoparticles (NPs) through exsolution has been widely explored.^29^ This method allows for the in situ growth of uniformly dispersed catalytically active NPs on the perovskite surface while the perovskite backbone acts as a stable support material.^30^

First described by Liu et al.,^31,32^ Sr_2_Fe_1.5_Mo_0.5_O_6_ (SFM) double-perovskite was studied as a redox-stable mixed ionic-electronic conducting material for symmetrical SOFCs. Various dopants at the B-site in SFM, including Ni,^33−37^ Co,^38−40^ Bi,^41^ Al,^41^ and Mg,^41^ for the exsolution of Fe- and Fe-based bimetallic NPs have been explored to enhance the performance of SFM-based electrodes, primarily for H_2_ fuel. Feng et al. reported improved stability and electrical conductivity for A-site-deficient Sr_1–*y*_Fe_1.4_Ni_0.1_Mo_0.5_O_6_ compared with the stoichiometric form (*y* = 0).^34^ Another study^37^ demonstrated that the tunability of electrochemical performance through Ni doping in Sr_2_Fe_1.5-x_Ni_*x*_Mo_0.5_O_6_ affected the exsolved Ni–Fe NPs. In recent studies, multiple research groups reported the cocatalytic impact of Ni–Fe alloys on ammonia conversion, leveraging the distinct metal-to-nitrogen binding energies of nickel and iron.^42,43^ However, despite the multifunctional nature of SFM as a promising material for symmetrical cells, encompassing redox stability and the exsolution potential of Ni–Fe alloys, its application as an electrode in DA-SOFCs has not been previously explored. In the field of cells directly fed with ammonia fuel (DA-SOFCs), Xiong et al. synthesized a Pr_0.6_Sr_0.4_Co_0.2_Fe_0.75_Ru_0.05_O_3-δ_ electrode with the exsolved CoFeRu-based alloy.^44^ Their electrolyte-supported cell reached a power density of 374 mW/cm^–2^ at 800 °C fed by NH_3_ fuel. Superior power densities were reported by He et al. through the exsolution of precious metallic Pd from a proton-conducting perovskite oxide electrode.^45^ Song et al. reported the decoration of La_0.55_Sr_0.30_TiO_3−δ_ (LST) perovskite with the exsolved Ni–Co NPs in DA-SOFC with improved performance compared to undoped LST.^46^ Recent studies have also reported enhanced electrochemical performance of perovskite oxide anodes with exsolved metallic NPs for NH_3_ fuel.^47,48^ However, despite their immense potential, research on perovskite-based electrocatalysts for NH_3_ fuel has lagged behind that on H_2_ and hydrocarbon-fed cells. Moreover, avoiding high-cost catalysts such as Ru, Pt, and Pd for high-performance DA-SOFC and the scaleup from laboratory size (<1 cm^2^) to commercial-grade cells are key factors for their widespread use. Additionally, to the best of our knowledge, the effects of exsolved FeNi_3_ NPs on direct-ammonia-fed SFM anodes have not yet been studied. Therefore, synthesizing robust and efficient perovskite-based electrodes for DA-SOFCs is of significant scientific and practical interest.

The present study focused on fabricating a new large-area DA-SOFC with advantageous high anode activity to ADR and HOR, long-term performance durability, and symmetrical configuration, enabling easy sintering and structural stability. Such parameters were achieved using A-site-deficient Sr_1.9_Fe_1.4_Ni_0.1_Mo_0.5_O_6_ (SFNM) double-perovskite as an anode material for DA-SOFC. The enhanced electrocatalytic activity toward ADR is achieved through the exsolved NiFe_3_ NPs. The chemical and physical stabilities of the SFNM under an ammonia atmosphere at temperatures up to 800 °C were determined. Subsequent morphology, chemical composition, and crystal structure of *in situ* exsolved Ni–Fe NPs were analyzed. The electrochemical activity of the SFNM-based electrodes for H_2_ and NH_3_ fuels was studied in a symmetrical electrolyte-supported cell with 3D-printed commercial-grade area (16 cm^2^) SFNM-GDC electrodes. This study demonstrated superior overall cell performance and long-term stability through j–*V*–*P* characterization. Finally, the mechanism of NH_3_ utilization over the SFNM-GDC electrode was investigated. This study highlights the beneficial impact of exsolved FeNi_3_ NPs on enhancing the efficiency of ammonia conversion, thereby improving the catalytic activity of the ADR, long-term stability, and power output. Furthermore, this anode microstructure engineering strategy promotes the potential adoption of DA-SOCs for commercialization as a clean energy conversion technology.

## Results and Discussion

### Characterization of the SFNM Electrocatalyst

**Figure 1:**
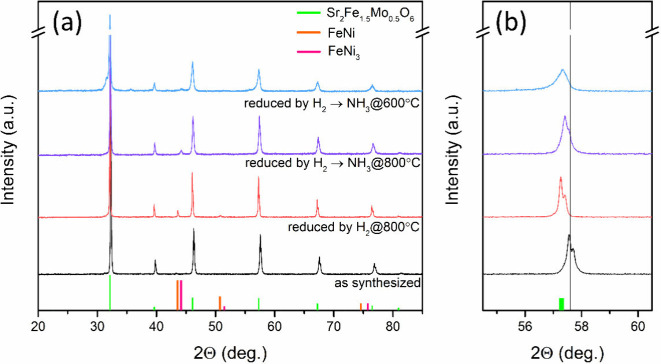
XRD patterns of SFNM powder: air calcinated, reduced by humidified H_2_ at 800 °C for 20 h, and reduced by humidified H_2_ at 800 and 600 °C for 20 h with exposure to NH_3_ for additional 3 h. (a) Full 2Θ spectra, (b) enlarged 2Θ area.

A phase analysis of the SFNM powder is shown in Figure 1. Evaluation of the air-calcinated SFNM revealed a single-phase cubic perovskite (a = 7.872 Å, *Fm*3̅*m*, PDF number 01–072–6388), identical to its Sr_2_Fe_1.5_Mo_0.5_O_6_ parent. This observation suggests that moderate Sr site deficiencies and partial substitution of Fe by Ni did not affect the formation of a single-phase cubic perovskite structure. However, a slight shift in the diffraction peaks toward higher angles was observed for the as-synthesized sample of the Sr_2_Fe_1.5_Mo_0.5_O_6_ (Figure 1b). This shift is likely attributed to lattice shrinkage resulting from the successful incorporation of the smaller ions of Ni^2+^ (0.72 Å) into Fe^2+^ (0.76 Å) sites in the perovskite lattice, as supported by previous studies.^37,49^ After 20 h of reduction in a humidified H_2_ (3% vol. H_2_O) atmosphere at 800 °C, several diffraction peaks corresponded to metallic Ni–Fe, indicating the formation of the cubic FeNi phase. These peaks were consistent with the decoration of the reduced samples with exsolved NPs.^35^ After reduction, the peaks of the parent SFM shifted back to lower angles while preserving the perovskite structure, highlighting its excellent redox stability.^32^

Similarly, a study of SFNM stability was conducted in an NH_3_ atmosphere at the operational temperatures of fuel cells using XRD. The procedure involved subjecting the samples to a reduction in humidified H_2_ at 800 °C for 20 h, followed by exposure to 20% NH_3_ (80% Ar as a carrier gas) for an additional 3 h at both 800 and 600 °C representing the standard operational temperature range for ceramic fuel cells. At high operating temperatures, the XRD pattern of the perovskite phase showed no new diffraction peaks beyond the original pattern (Figure 1a). Notably, Ni enrichment in the exsolved NPs indicated the formation of a cubic FeNi_3_ phase. When exposed to NH_3_ at a low operating temperature (600 °C), an additional diffraction peak emerged at 31.52°.

This peak might suggest the formation of an active Ruddlesden–Popper phase Sr_3_Fe_2-x-y_Ni_*x*_Mo_*y*_O_7-δ_.^50^ However, no evidence was available for the nitriding of different phases within the tested temperature range of 800 and 600 °C. These findings highlight the robust chemical stability of the SFM in an NH_3_ environment, which is crucial for its effectiveness as a fuel electrode in DA-SOFCs.

**Figure 2:**
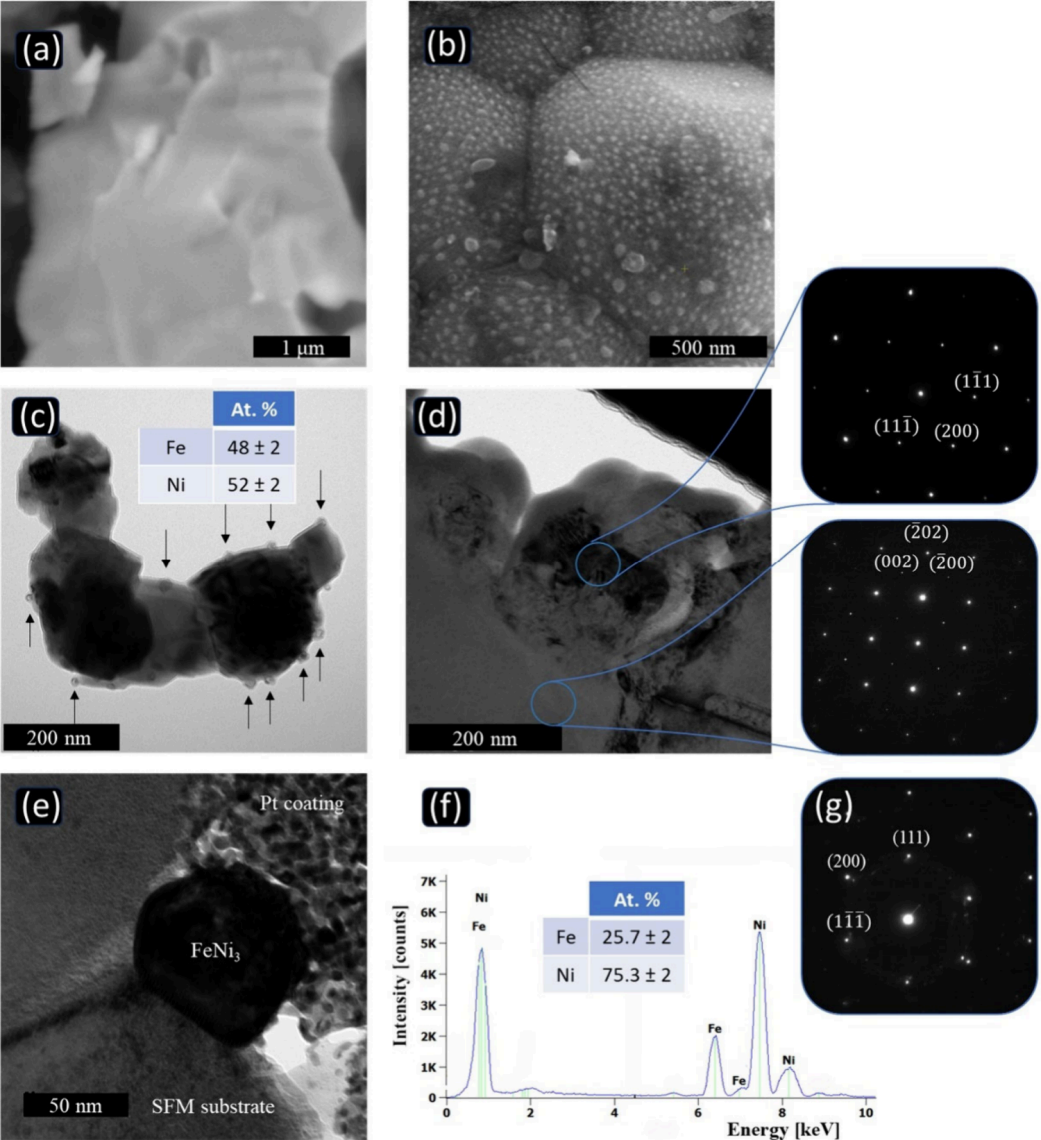
SEM images of the (a) air-sintered SFNM pellet. (b-c) SFNM pellet and powder after reduction at 800 °C under humidified H_2_ (3% H_2_O) and corresponding TEM cross-sectional image. (d) TEM cross-sectional image with SAED patterns taken from the substrate and the exsolved NPs at the [011] and [010] zone axes, respectively. (e) Cross-sectional TEM image of the sample after exposure to NH_3_. (f) EDS spectrum and (g) SAED pattern taken from the NP shown in (e) at the **[011̅]** orientation. Additional spots originate from Pt coating added on top of the NP as part of the FIB sample preparation.

Further morphological changes and NP exsolution investigations were conducted using electron microscopy. The perovskite powder underwent pelletization, sintering, and subsequent reduction in humidified H_2_. The morphology of the synthesized SFNM pellet after 3 h of sintering at 1200 °C in air revealed a clean and uniform grain surface (Figure 2a). After reduction with H_2_, the *ex-situ* SEM image showed evenly spread nanometrically exsolved species (Figure 2b). The reduced SFNM surface contained nearly spherical particles with an average size of 40–200 nm. These NPs were considered to be exsolved metallic Ni–Fe with an almost 1:1 Ni:Fe ratio, as confirmed by the average elemental analysis of several NPs (Figure 2c), and correlate with the XRD diffraction pattern of the sample before exposure to NH_3_.

Furthermore, TEM analysis of the cross-sectional lamellae validated the local elemental composition and crystal structures. The SAED pattern of the SFM substrate was indexed in terms of Sr_2_Fe_1.5_Mo_0.5_O_6_ (a = 7.872 Å, *Fm*3̅*m*), matching the XRD analysis results and providing additional support for the phase stability of the SFNM under reducing conditions. The SAED pattern obtained from the crystalline Ni–Fe NPs was indexed as Ni_*x*_Fe_1–*x*_,^51^ consistent with the EDX measurements of the NPs, as shown in Figure 2d. After exposure to an NH_3_ atmosphere, the composition of the NPs approached a 1:3 ratio (i.e., FeNi_3_), as depicted in Figures 2e-g. The successful indexing of the SAED pattern confirms that the crystal structure of the particles remained cubic, with the Fm3̅m space group similar to conditions with the absence of ammonia.

The electrocatalytic activity of the double-perovskite Sr_1.9_Fe_1.4_Ni_0.1_Mo_0.5_O_6_ (SFNM) is strongly influenced by the multivalency of B-site elements (Fe, Mo, and Ni) and the oxygen nonstoichiometry (6-δ) values. Evaluation of the elemental compositions and the ratio of various valency states of the B-site components in SFNM under actual operating conditions was performed using X-ray photoelectron spectroscopy (XPS) analysis.

The wide spectra of SFNM pellets initially calcined in air, subsequently reduced in dry H_2_, and finally exposed to NH_3_ at 800 and 600 °C are shown in Figure S1. Peaks corresponding to Sr 4p, Fe 3p, Mo 3d, Sr 3p, Sr 3s, O 1s, Fe 2p, N 1s, and Ni 2p, along with Auger peaks at high binding energies (>940 eV), were identified and matched with the elemental composition of the SFNM using the NIST XPS database.^52^ Peaks associated with carbon (C 1s at 286 eV) were also observed and served as a reference for the peak shifts.

**Figure 3:**
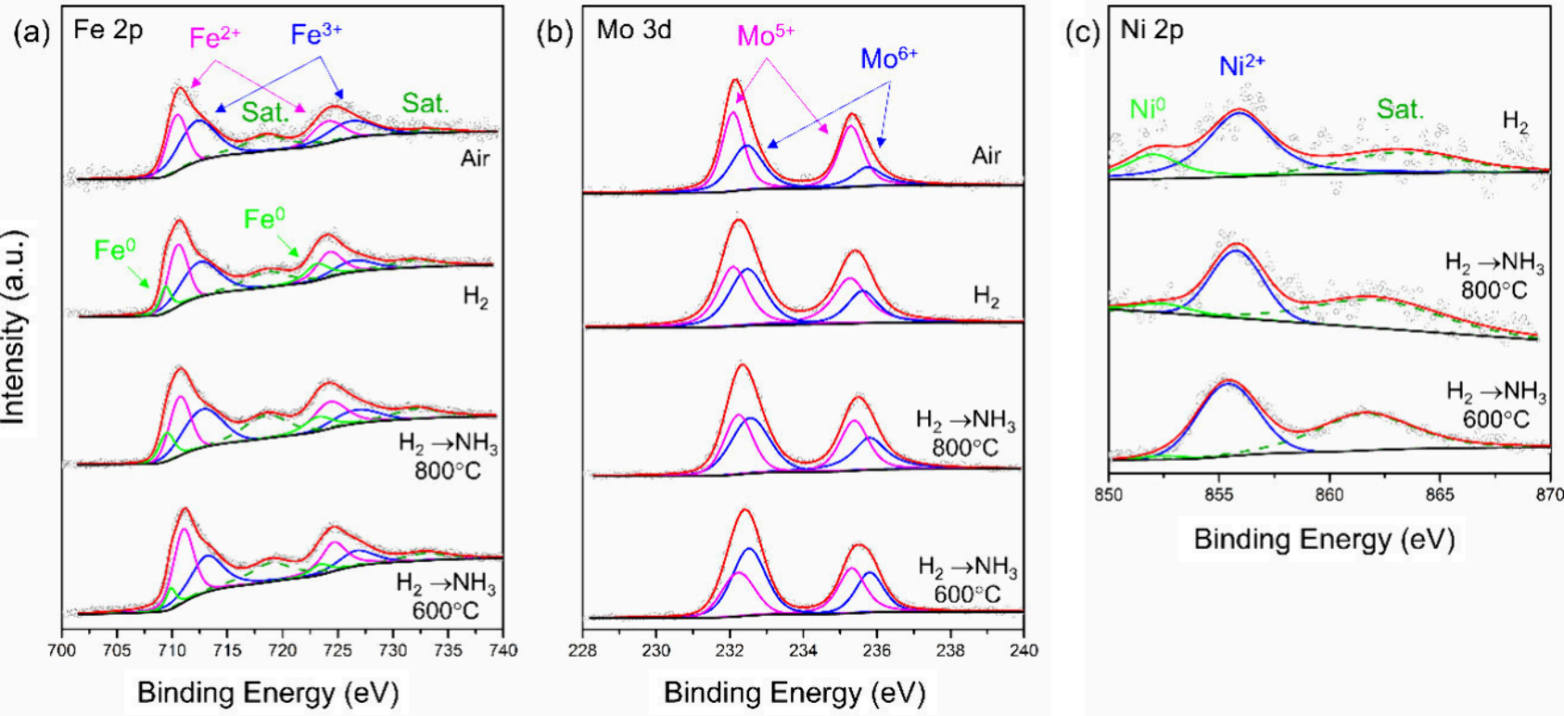
XPS spectra for (a) Fe 2p, (b) Mo 3d, and (c) Ni 2p species in SFNM pellets after air calcination, reduction by H_2_, and exposure to NH_3_ at 800 and 600 °C.

The XPS analysis in Figure 3a revealed the appearance of the Fe 2p core-level spectra of the four examined samples. Upon reduction in H_2_, the SFNM surface reveals peaks corresponding to metallic Fe^0^ with peak positions of 723.2 and 709.6 eV for Fe^0^2p_3/2_ and Fe^0^2p_1/2_, respectively. The calculated Fe^0^/F*e*_total_ ratio was 0.20 for SFNM reduced in H_2_ and decreased to 0.18 and 0.12 under exposure to NH_3_ at 800 and 600 °C, respectively. Additionally, the Fe 2p deconvolution revealed the presence of mixed valence states with a redox couple of Fe^2+^ and Fe^3+^. The positions of Fe^2+^ 2p_1/2_ and Fe^2+^ 2p_3/2_ peaks were identified at 724.3 and 710.6 eV (±0.2 eV), whereas those of Fe^3+^2p_3/2_ and Fe^3+^2p_1/2_ peaks were at 726.5 and 712.7 eV (±0.2 eV), respectively.^53^ The doublet structure with a difference in B.E. of ∼13.6 eV confirmed the presence of Fe 2p_1/2_ and Fe 2p_3/2_ doublets in each SFNM sample. The calculated Fe^2+^/Fe^3+^ ratios were 0.68, 0.92, 1.01, and 1.02 for as-synthesized SFNM (air), reduced in H_2_ and exposed to NH_3_ at 800 °C and at 600 °C, respectively.

Similarly, Figure 3b shows the deconvolution of the Mo 3d core-level spectra. The peaks corresponding with Mo^6+^3d_5/2_, Mo^6+^3d_3/2_ (232.5 and 235.7 eV), and Mo^5+^3d_5/2_, Mo^5+^3d_3/2_ (232 and 235.2 eV)^40,54^ were observed, and the Mo^5+^/Mo^6+^ ratios were calculated as 1.53, 1.17, 0.91, and 0.79 in as-synthesized SFNM (air), reduced in H_2_, and exposed to NH_3_ at 800 °C and that at 600 °C, respectively. The opposite trends in the Fe^2+^/Fe^3+^ and Mo^5+^/Mo^6+^ ratios suggest that Fe^2+^ acts as a charge carrier, whereas the Mo^6+^ concentration increases during charge compensation and stabilization under reducing conditions.

Furthermore, Figure 3c showcases the deconvolution peaks of Ni 2p in reduced SFNM and samples exposed to NH_3_ at 800 and 600 °C. Three peaks corresponding to Ni^2+^ (855.7 eV ± 0.2 eV), metallic Ni^0^ (852 eV ± 0.25 eV), and a satellite peak of Ni (862.2 eV ± 0.7 eV) were observed.^54^ The Ni^0^/Ni^2+^ ratios were estimated as 0.35, 0.25, and 0.06 in reduced SFNM, exposed to NH_3_ at 800 °C and at 600 °C, respectively. These trends suggest that the exsolution of Ni is strongly influenced by H_2_ partial pressure and temperature, with high temperatures favoring Ni exsolution. The occurrence of metallic Fe^0^ and Ni^0^ species under anodic conditions corresponds to the exsoluted FeNi_3_ previously indicated by the XRD analysis.^55^ However, this observation implies that complete exsolution of Fe and Ni from the parent oxide may not be achievable, as previously suggested.^56^ Additionally, ADR can be segmented into three consecutive stages: (1) adsorption of ammonia molecules onto the catalyst’s active sites, (2) cleavage of N–H bonds on ammonia molecules, and (3) recombination and desorption of nitrogen atoms.^57^ Therefore, chemisorbed nitrogen species from the catalytic reaction could account for the change in Ni^0^ content, while catalyst nitriding is typically observed at lower operating temperatures, coinciding with reduced ammonia decomposition rates.^24^

In addition, the O 1s spectra (Figure S2a) revealed three peaks at 529.8, 530.9, and 532 eV, which can be attributed to lattice oxygen, oxygen defects, and surface-adsorbed oxygen species, respectively.^58−60^ The percentage area under the deconvoluted peaks (O_lat_.:O_v_:O_Ads_.) shown in Table 1 varied with the operating conditions, indicating changes in the oxygen vacancy (O_v_) and adsorption (O_Ads_.) compared to the lattice oxygen (O_lat_.). Under reducing conditions, the concentrations of O_v_ and O_Ads_. species significantly increased, and subsequent exposure to NH_3_ at 800 and 600 °C led to a minor reduction in their partial amount. The exsolved Ni^0^ on the surface of the reduced SFNM may also affect the ratio trends between the oxygen species under various operating conditions. In summary, the SFNM shows promise as an electrocatalyst, exhibiting activities associated with oxygen transport properties under the operating conditions of DA-SOFCs.

**Table 1:** Notable Trends in SFNM Unveiled through Deconvolution of XPS Spectra

Atmosphere					O_lat_.:O_v_:O_Ads_	
Air	0.68	1.53	---	---	0.47:0.26:0.27	---
H_2_	0.92	1.17	0.20	0.35	0.21:0.48:0.31	0.47
NH_3_ @ 800 °C	1.01	0.91	0.18	0.25	0.20:0.48:0.32	0.43
NH_3_ @ 600 °C	1.02	0.79	0.12	0.06	0.22:0.48:0.30	0.39

Finally, the N 1s spectra (Figure S2b) revealed three peaks at 397.5 eV, 398.3, and 400.3 eV. These peaks may be assigned to the adsorbed nitrogen linked to metallic species (N-M^0^) and metal cations, such as Fe^2+/3+^ and Ni^2+^ (N-M^(+)^), and oxygen (N–O), respectively.^61,62^ Notably, metallic species, such as exsolved nanoparticles, are responsible for 47%, 43%, and 39% of the adsorption nitrogen in reduced SFNM, exposed to NH_3_ at 800 and 600 °C, respectively. This underscores the potential of exsolved FeNi_3_ NPs as active surfaces for ADR.

The XPS analysis indicated the presence of mixed valence states of the B-site elements Fe and Mo and the exsolution behavior of Ni in the SFNM under various operating conditions. The Fe^2+^/Fe^3+^ and Mo^5+^/Mo^6+^ ratios highlighted the role of Fe^2+^ as a charge carrier and the stability-enhancing properties of Mo^6+^. The in situ exsolution of FeNi_3_ NPs under DA-SOFC conditions, influenced by temperature and ammonia exposure, supports the application of SFNM as a promising oxide electrocatalyst for enhanced ADR/HOR activities with improved stability under reducing conditions.

### Characterization of the Symmetrical Electrolyte-Supported Cell

**Figure 4:**
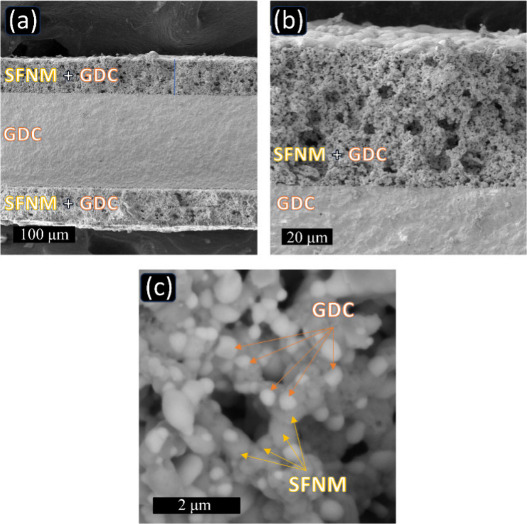
DIW 3D-printed symmetrical SOFC with SFNM electrodes and tape-casted GDC electrolyte; (a) image of a cross-section, and (b-c) enlarged SEM images.

The direct ink writing (DIW) 3D printing method was selected for fabricating the SFNM-GDC electrodes because of its flexibility, fine resolution control, and suitability for producing large-area ceramic cells.^63^Figure 4a shows the GDC-supported cell’s cross sections with 3D printed SFNM-GDC electrodes with a thickness of ∼50 μm. As shown in Figure 4b, outstanding connectivity was observed between the fuel cell components at the interface.

The examination of the morphology of the electrode revealed the development of micro- and submicropores following the combustion of organic media, along with the emergence of mesoporosity upon the removal of the PMMA pore former (Figure 4b and 4c). The well-connected pore network is advantageous for gas diffusion and aids the reduction process at the anode, facilitating fuel diffusion toward the reactive sites on the electrode.^64^ Furthermore, an even distribution of SFNM and GDC was observed, establishing a conjugated structure and enhancing the density of the triple-phase boundary (TPB) sites.

### Electrochemical Performance of the Cell Using H_2_ and NH_3_ Fuels

**Figure 5:**
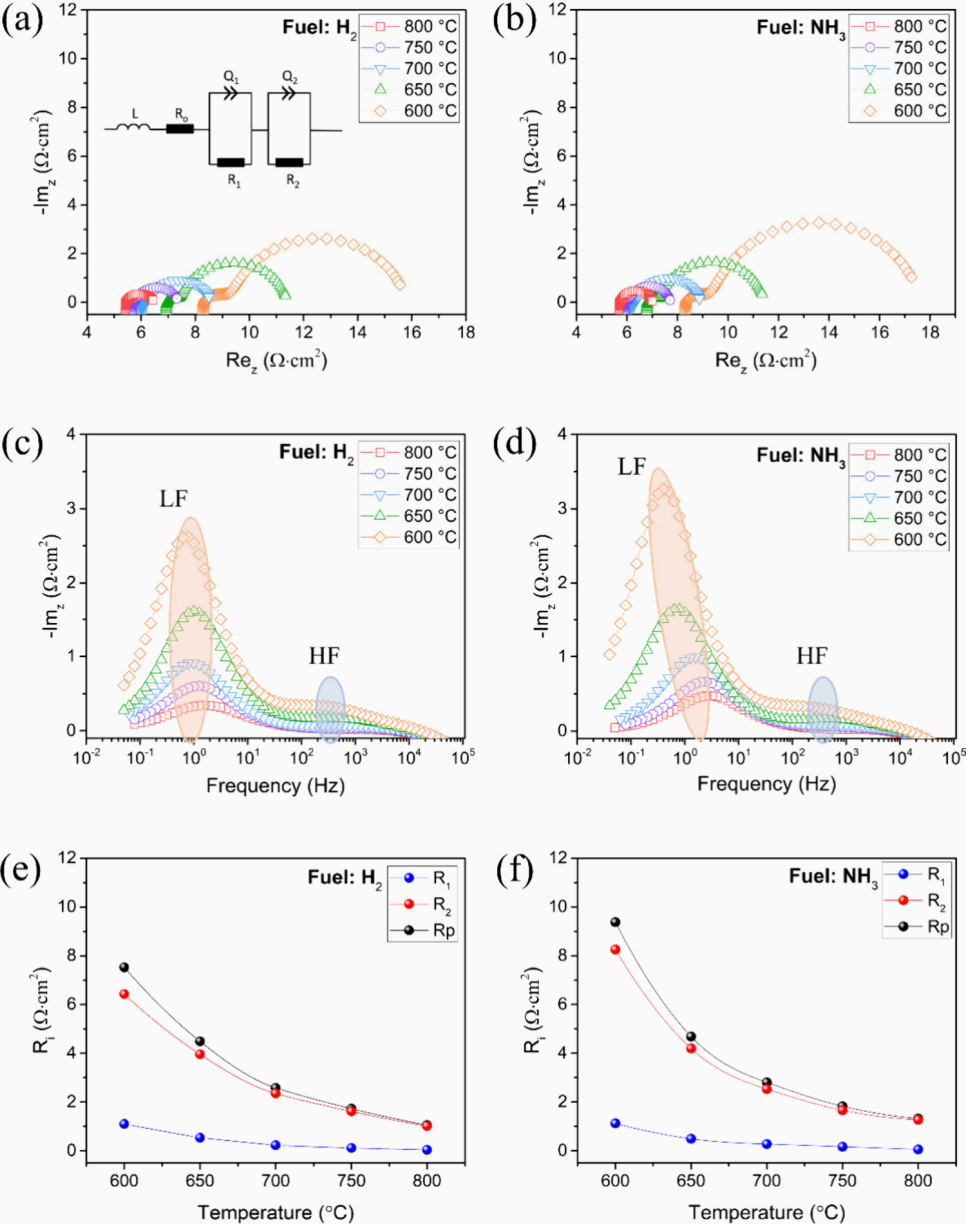
EIS analysis of the symmetrical half cell. Nyquist plots obtained at 600–800 °C, corresponding Bode plots, and temperature-dependent variation in *R*_i_. Images (a), (c), and (e) are for H_2_ fuel and (b), (d), and (f) are for NH_3_, respectively.

Nyquist plots representing the electrochemical performance of the synthesized symmetrical cell for H_2_ and NH_3_ fuels are shown in Figures 5a and 5b, respectively. The EIS spectra were analyzed using an *LR*_0_*(Q*_1_*/R*_1_*)(Q*_2_*/R*_2_*)* equivalent circuit, as shown in Figure 5a. In this model, *L* is the inductance, *R*_*o*_ is the ohmic resistance, primarily associated with the electrolyte and the setup ohmic resistance, and *Q*_*i*_*/R*_*i*_ are the Z_arc_ elements used to determine the various interfacial electrochemical activities during the anode reaction. Anomalous capacitance dispersion was observed at the interface between the two solid layers in solid electrode systems. This behavior is described by an equivalent circuit using a parallel configuration of the constant-phase element *Qi* and resistance *Ri*. The polarization resistance (Rp) was determined by analyzing the intercept of the impedance arc with the real axis at high frequencies (HF) and low frequencies (LF).

Two distinct kinetic processes governing the electrochemical reaction are revealed by the Bode plots for the H_2_ and NH_3_ fuels, as illustrated in Figure 5c and 5d, respectively. The first peak, attributed to *R*_1_, appeared in the HF region at approximately 10^3^ Hz and was attributed to charge transfer at the electrode, as supported by Miyazaki and Muroyama.^65^ The second, attributed to *R*_2_, was recognized in the LF region at approximately 1 Hz and addressed as a mass transfer phenomenon encompassing fuel conversion, diffusion, and adsorption processes. The values of *Ri* for each subprocess of the anode reaction are shown in Table S1, and their temperature-dependent variations are depicted in Figure 5**e and**5**f**. Here, the resistances (*R*_1_ and *R*_2_) of both gases exhibited a similar trend, decreasing with increasing temperature, signifying thermodynamically active phenomena. Within the 700–800 °C temperature range, the SFNM-GDC electrode demonstrated low and nearly equal polarization resistances, indicating favorable activity for HOR and ADR.

Notably, at relatively low operating temperatures, the *R*_2_ resistance of NH_3_ was more pronounced than that of H_2_. This behavior may be attributed to the diminished decomposition rate of ammonia at low temperatures, resulting in a decrease in H_2_ content. The resemblance in the shapes and positions of LF peaks for H_2_ and NH_3_ above 700 °C, as depicted in Bode plots, suggests the involvement of identical species, such as hydrogen, in the electrochemical reaction. This observation was consistent with the findings of Zhong et al.^47^ This implies that NH_3_ undergoes a decomposition reaction to form N_2_ and H_2_, which is subsequently oxidized via the HOR. However, in contrast to the H_2_-fed cell, peaks at the LF for NH_3_ below 700 °C shift to a lower frequency range (<1 Hz), attributed to gas conversion impedance.^66^ This observation further supports the dominant contribution of ammonia conversion to the mass transport resistance at low operating temperatures. Despite the favorable charge transfer process, the anode reaction is mainly dominated by the mass transfer phenomenon, accounting for 85.4–96.2% and 88.0–96.2% of the polarization resistance for H_2_ and NH_3_, respectively. It can be inferred that mass transfer resistance plays a predominant role in the HOR and ADR, serving as the rate-determining step.

Finally, the activation energy for each interfacial reaction process was evaluated as a key factor affecting the electrode activity. The Arrhenius plots (Figure S3) demonstrated through linear fitting that all the interfacial reaction processes were thermally activated. As depicted in the figure, the Ea values for the charge transfer (R_1_), mass transfer (R_2_), and polarization resistance (Rp) were 1.39, 0.82, and 0.87 eV for H_2_ and 1.23, 0.84, and 0.87 eV for NH_3_, respectively. The similarity in the activation energy between the H_2_ and NH_3_ fuels indicates the high suitability of SFNM as a catalyst material for DA-SOFC anodes.

### Ammonia Conversion, Cell Performance, and Reaction Mechanism of the Ammonia-Fueled Anode

**Figure 6:**
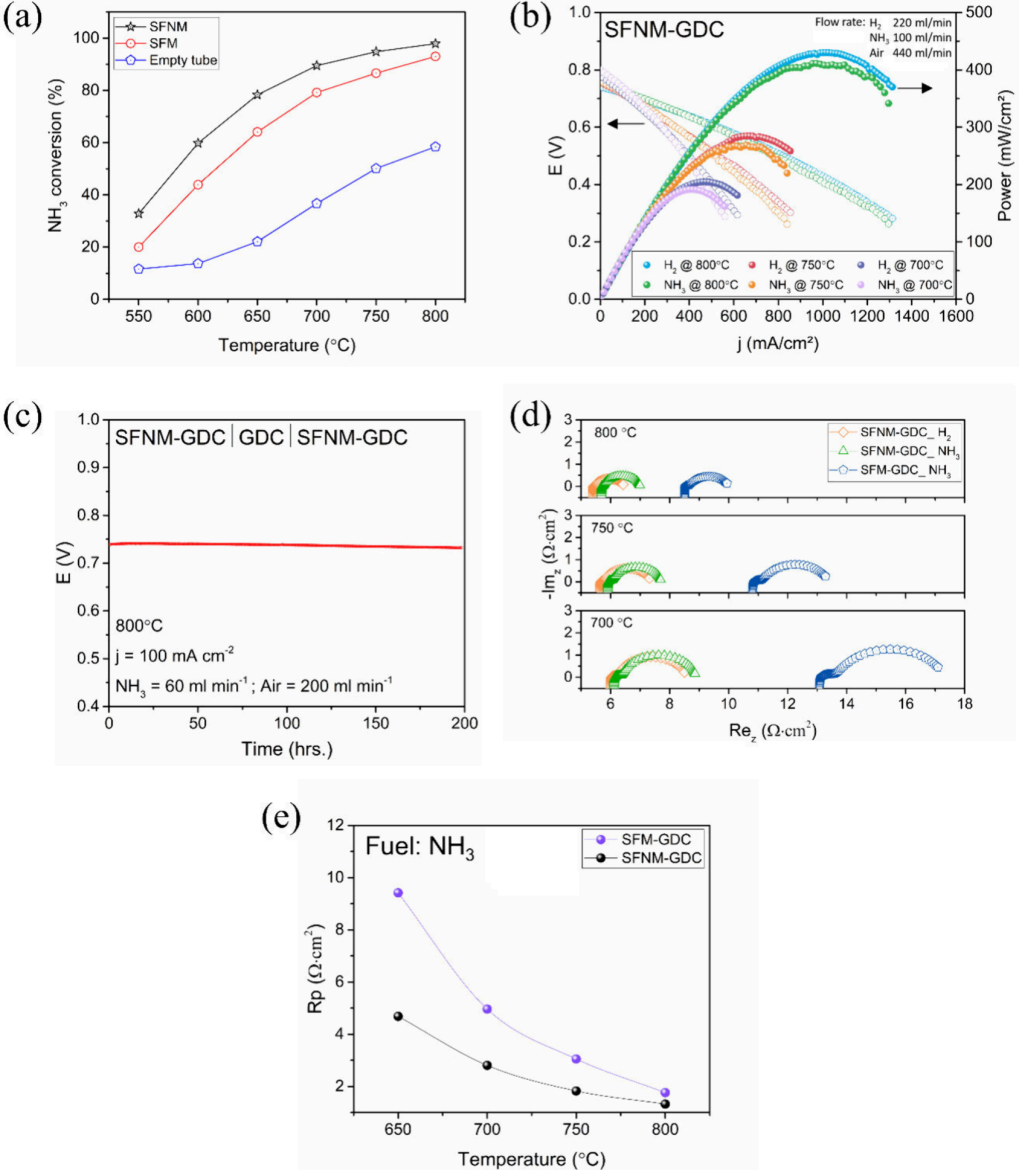
Comprehensive characterization of the SFNM anode. (a) Ammonia decomposition curves. (b) *j*–*V*–*P* characterization. (c) Long-term operation of the symmetrical fuel cell with 200 μm electrolyte and SFNM-GDC electrodes. (d) Nyquist plots of symmetrical half cells with SFNM-GDC and SFM-GDC electrodes. (e) Temperature-dependent variation in polarization resistance.

An illustration of the robust catalytic activity of SFNM for ADR in DA-SOFC anodes is shown in a comprehensive comparison with SFM (Sr_1.9_Fe_1.5_Mo_0.5_O_6_) and an empty quartz tube, which served as a control for eliminating the self-decomposition of ammonia. As depicted in Figure 6a, the reduced SFNM displayed superior catalytic activity for NH_3_ conversion compared with the undoped SFM. Specifically, at operating temperatures of 800 °C, 750 °C, and 700 °C, SFNM exhibited conversion rates of 97.9%, 94.8%, and 89.5%, respectively, outperforming the rates of 93.0%, 86.6%, and 79.2% observed for SFM. This enhanced activity of the SFNM was attributed to the improved reaction kinetics induced by the presence of highly active-exsolved FeNi_3_ nanoparticles.

The *j*–*V*–*P* characterization of cells with H_2_ and NH_3_ as fuels and air as oxidant gas is depicted in Figure 6b. Examination of the symmetrical cell with identical SFNM-GDC electrodes under operation with H_2_ revealed peak power density (PPD) values of 430, 286, and 205 mW cm^–2^ at 800, 750, and 700 °C, respectively. For cells operated by NH_3_ fuel, PPD values of 416, 270, and 193 mW cm^–2^ were recorded at 800, 750, and 700 °C, respectively. This behavior indicates an exceptionally minor reduction in fuel cell performance upon fuel substitution (only 3.5% at 800 °C). Furthermore, the PPD values achieved with NH_3_ are noteworthy compared to those of different perovskite-based electrodes with exsolved Ni, NiCo, and even Ru-based NPs.^44,46,47^ Additionally, when operated with NH_3_ fuel, the cell demonstrated excellent long-term stability in galvanostatic mode, applying a constant current of 100 mA cm^–2^ (Figure 6c). The cell demonstrated an average voltage loss of −4.7 × 10^–5^ V h^–1^, corresponding to a degradation rate of about 0.48% per 100 h.

To gain a deeper understanding of the proposed operational mechanism of the SFNM as an active material for NH_3_ fuel, several cells were fabricated with SFM-GDC electrodes while keeping the other cell components and fabrication procedures identical. Within this configuration, the PPD decreased for both fuels, measuring 351 and 305 mW cm^–2^ at 800 °C for H_2_ and NH_3_, respectively (Figure S4). This resulted in a 13.1% degradation in power output upon switching fuels. Compared to the low PPD degradation observed in SFNM-GDC when switching fuels, the positive impact of the exsolved FeNi_3_ NPs on the electrode performance when utilizing NH_3_ as a fuel becomes evident. Figure 6d presents Nyquist plots of symmetrical half-cells employing SFNM-GDC (SFNM with exsolved NPs) and SFM-GDC (pristine SFM) electrodes. The corresponding temperature-dependent evolution of polarization resistance is shown in Figure 6e. As explained earlier, the increased ohmic resistance observed in the presence of NH_3_ fuel was attributed to the temperature decrease resulting from the endothermic NH_3_ decomposition process. Furthermore, the predominant increase in the fuel concentration resistance, which subsequently led to elevated polarization resistance (Rp) values, underscores the sluggish rate of ammonia decomposition in the absence of FeNi_3_ NPs. These findings emphasize the importance of FeNi_3_ NPs as active surfaces for ammonia decomposition, thereby improving cell performance.

As explained in Section 1, NH_3_ utilization in DA-SOFC involves multistep ammonia decomposition followed by HOR (R1 and R2) reactions. However, a competitive single-step reaction path for NH_3_ oxidation is also possible, as outlined in Reaction R4:
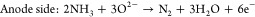
R4For the SFNM-GDC electrode, the similarity in cell performance for both fuels suggests that the dominant pathway might be a multistep reaction, with the exsolved FeNi_3_ NPs serving as active catalysts for the ADR. Consequently, the difference in the PPDs of H_2_ and NH_3_ can be attributed to the local temperature decrease on the electrode surface caused by the endothermic nature of ADR, coupled with the dilution effect due to the formation of inert N_2_ as a byproduct.^67^

An additional approach to elucidate the reaction pathway of NH_3_ on the anode involves analyzing the variation in the open-circuit voltage (OCV) with the cell’s operating temperature.^68,69^ The temperature dependence of the standard Gibbs free energy of the HOR and direct NH_3_ oxidation showed contrasting trends. The theoretical OCV at each temperature was calculated using Equation 1, where *n* represents the number of electrons involved in the chemical reaction, and *F* is the Faraday constant.
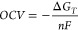
1As shown in Table 2, when H_2_ is supplied to the anode, the theoretical OCV decreases with increasing temperature. However, the theoretical values of the OCV in the route of NH_3_ direct oxidation exhibited the opposite trend. If the ammonia reaction pathway involves the initial decomposition of NH_3_ into H_2_ and N_2_, followed by the HOR, one would expect the variation in the OCV to match the pattern observed for the H_2_ fuel. Considering these theoretical expectations, the SFNM-GDC electrode demonstrated the same trend when fed with H_2_ and NH_3_ fuels. These results further support that NH_3_ is primarily utilized in the dual-step routes for the SFNM-GDC electrodes. However, cells fed with both fuels exhibited lower experimental OCV values than the theoretical calculations, which may be attributed to the mixed ionic-electronic conduction of the doped-ceria electrolytes.^70^

**Table 2:**
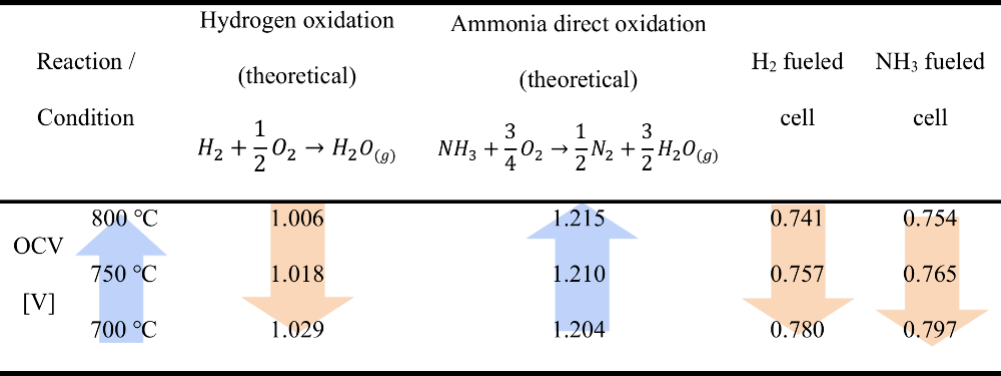
Theoretically Calculated and Experimental OCV Values for H_2_ and NH_3_ Fueled Cells

**Figure 7:**
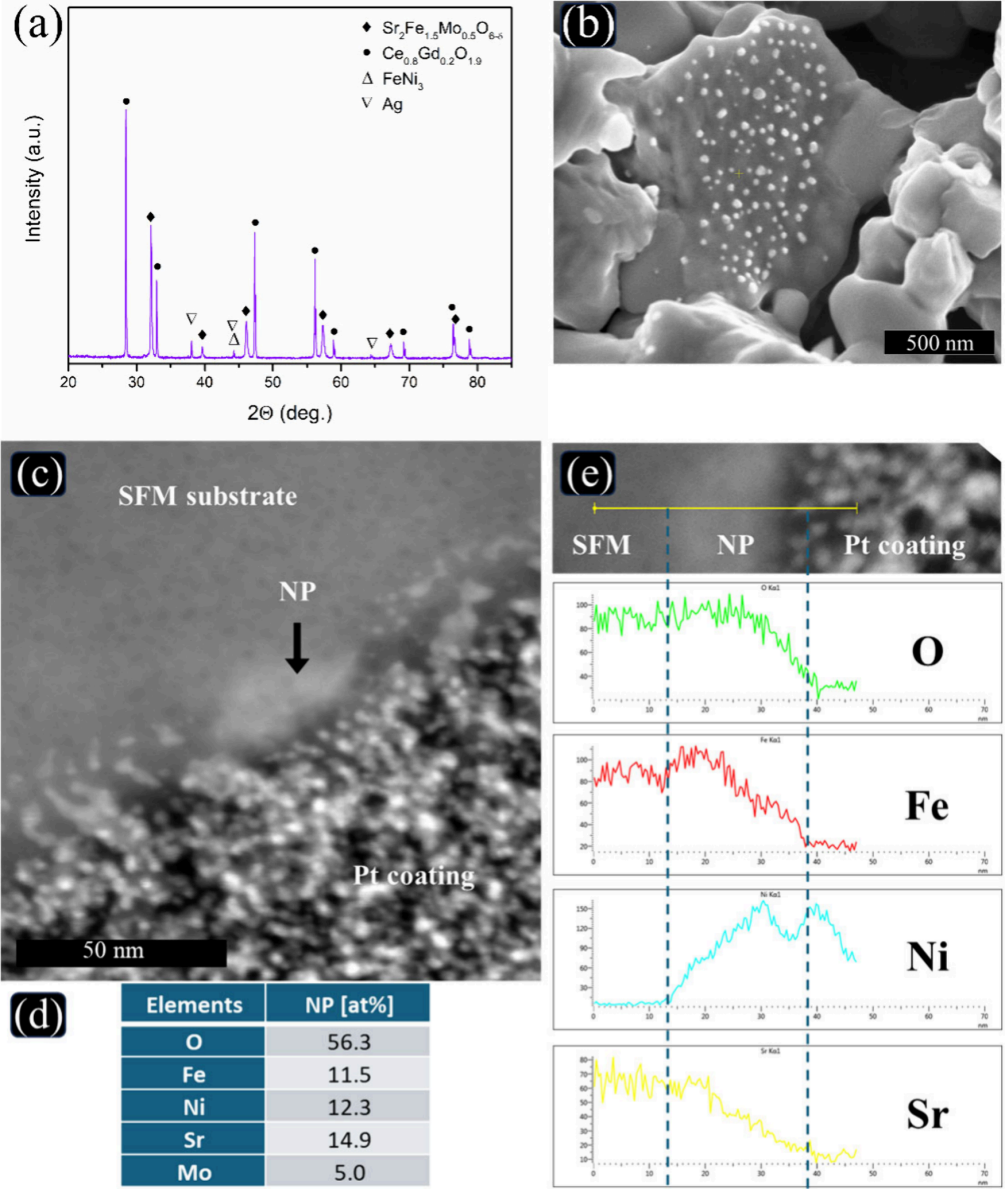
Post-mortem analysis of SFNM-GDC electrode after 200 h of galvanostatic operation. (a) Phase analysis and (b) SEM cross-sectional image of the electrode. (c) cross-sectional scanning TEM image of the electrode. (d) EDS spot analysis is taken from the NP. (e) Line scan EDS analysis (yellow line on the upper image is 70 nm). For clarity, the area of the particle is marked by dashed lines.

A post-mortem analysis of the ammonia-fed cell, conducted after 200 h of galvanostatic operation, was performed to further assess the stability of the SFNM-GDC electrode. Figure 7a shows that phase analysis reveals the primary phases in the electrode are Sr_2_Fe_1.5_Mo_0.5_O_6_ and Ce_0.8_Sm_0.2_O_1.9_. Additionally, metallic phases of FeNi_3_ and silver (Ag) are identified, corresponding to the exsolved NPs and the current collection layer, respectively. The absence of undesired parasitic phases further underscores the excellent phase stability of the electrode under extended operation. As shown in Figure 7b, exsolved NPs, ranging from 30 to 60 nm in size, are observed on the surface of the original SFM substrate. However, there is a quantitative reduction in the number of particles and a slight decrease in dispersion compared to the fresh electrode (shown in Figure 2). The corresponding TEM analysis, depicted in Figure 7**c-e**, illustrates compositional changes of the NPs after 200 h of galvanostatic operation. Although spot EDS shows the averaged composition of the NP as close to 1:1 (Ni:Fe) (Figure 7d), the line scan shown in Figure 7e, reveals compositional variations within the NPs in the sample, with the Ni ratio increasing further from the substrate. We believe that the addition of oxygen and Sr to the NP, which originates from the SFM substrate, should be excluded. Interestingly, nitrogen was not detected in the tested samples, indicating a facilitated nitrogen desorption step. This was achieved by the cocatalytic impact of the nickel and iron catalyst on ammonia conversion, stemming from the differing metal-to-nitrogen binding energies of the two elements.

Overall, the enhanced catalytic activity of the SFNM-based electrodes for NH_3_ conversion, along with the decreased polarization resistance and improved performance for HOR, can be attributed to the surface decoration of highly active FeNi_3_ nanoparticles. These FeNi_3_ nanoparticles facilitate NH_3_ decomposition reactions, which provide more hydrogen fuel for subsequent HOR at the anode, enabling greater power densities and long-term durability. Moreover, optimizing the Ni doping levels and exploring codopant strategies in the SFM double-perovskite could help increase the density and lower-temperature activity of exsolved NPs to improve ammonia conversion, reduce polarization losses, and enhance overall cell performance. Coupling these catalyst optimization approaches with electrode microstructural engineering and exploiting the symmetrical SOFC configuration for reversible operation could further advance direct-ammonia-fueled cells to support sustainable clean energy technologies.

## Conclusions

The superior performance and stability of SFNM perovskite-based electrodes in DA-SOFCs were successfully demonstrated. When exposed to ammonia, the SFM backbone exhibited remarkable phase stability throughout the exsolution process. The reduction of SFNM resulted in surface decoration with Ni–Fe NPs, which were further transformed into FeNi_3_ NPs upon exposure to ammonia. The active-exsolved FeNi_3_ NPs significantly enhanced the ammonia conversion rate within the operating temperature range of the cell, outperforming the reference tube and the bare SFM. The 3D-printed symmetrical cell, featuring SFNM-GDC electrodes, exhibited a PPD of 430 and 416 mW cm^–2^ for H_2_ and NH_3_ fuels, respectively, with confirmed long-term stability through testing. The electrodes exhibited excellent stability during extended galvanostatic operation under direct-ammonia fuel, with a degradation rate of 0.48% per 100 h. The ammonia consumption mechanism was identified as a multistep process involving ammonia decomposition followed by the HOR.

Further scientific pathways may be focused on controlling Ni concentration in SFM to enhance NH_3_ conversion, refining the anode microstructure and fabrication process to reduce Rp values, and leveraging the symmetrical SOFC structure for its reversible operation. In summary, the utilization of highly electrochemically active, reliable, and redox-stable SFNM-based electrodes in large-area DA-SOFCs represents significant progress toward the direct use of NH_3_ to replace H_2_ as a fuel.

## Methods

### Material Synthesis

SFNM was successfully synthesized via citric acid-assisted glycine-nitrate processing (GNP). Single-step synthesis was performed as follows: the stoichiometric amounts of Ni(NO_3_)_2_·6H_2_O (Spectrum Chemical Mfg. Corp., USA), Sr(NO_3_)_2_ (Thermo Scientific, USA), (NH_4_)_6_Mo_7_O_24_·4H_2_O (Alfa Aesar, USA), and Fe(NO_3_)_3_·9H_2_O (Thermo Scientific, USA) were dissolved in deionized water. Citric acid and glycine were added to a solution of metal ions in a molar ratio of 1.5:2:1. After 1 h of continuous stirring, the water evaporated at 80 °C until a dark gel was formed. The gel underwent a self-combustion process at 220 °C in a ventilated furnace, followed by a 5-h calcination at 1050 °C to achieve the final single-phase SFNM.

### Cell Fabrication

Electrolyte-supported symmetrical SOFCs were fabricated by DIW 3D printing of SFNM-GDC (Gd_0.2_Ce_0.8_O_2_) electrodes over a tape-cast rectangular 25 cm^2^ GDC electrolyte, as illustrated in Figure S5.

For the DIW printing of the SFNM-GDC electrodes, inks with ∼50% solid loading and an SFNM/GDC ratio of 60:40 were produced. First, the required amounts of the synthesized SFNM powder and commercial GDC (Fuelcellmaterials, USA) were dispersed in ethanol and roll-milled for 24 h with zirconia balls. After drying the ethanol, the milled powders were mixed by a centrifugal mixture with a terpineol-based vehicle (VEH, Fuelcellmaterials, USA), α-terpineol (Alfa Aesar, USA), and PMMA (pore former, Fiaxell, Switzerland) with a weight ratio of 15.6:15.6:2.4:1, respectively. The resulting mixture was vigorously mixed using a centrifugal planetary mixer until a homogeneous ink was obtained. A viscometer (Brookfield, MLVT115, USA) was used for viscosity measurements, evaluating a value of 31.1 Pa s, employing spindle number 64 at a shear rate of 60 rpm. The ink was printed using a self-modified 3D printer with an inner nozzle diameter of 0.97 mm. The nozzle-to-substrate (GDC layer) distance and flow rate were set at 0.1 mm and 0.18 mm^3^ mm^–11^, respectively. Subsequently, symmetrical SFNM-GDC electrodes with an area of 16 cm^2^ were fabricated, followed by multistep cosintering up to 1200 °C for 3 h to form a symmetrical SFNM-GDC|GDC|SFNM-GDC fuel cell.

### SFNM Characterization

Phase analysis of the synthesized SFNM powder was performed using a SmartLab SE diffractometer (Rigaku, Japan) at Bragg–Brentano geometry with copper anode tube, at 40 kV and 30 mA, and a scanning rate of 2.5° min^–1^. SFNM pellets were analyzed by XPS using an ESCALAB 250 spectrometer (Thermo Fisher Scientific, USA) equipped with an Al K_α_ X-ray radiation source under ultrahigh vacuum conditions. The core-level spectra of Fe, Mo, Ni, N, and O in the SFNM lattice and the exsolved nanoparticles were analyzed by deconvolution using XPSPEAK41 software to elucidate the catalytic activity of SFNM under DA-SOFC operating conditions.

Microstructural analysis of the electrodes and exsolved NPs was performed using a Helios 4G UC-focused ion beam scanning electron microscope (Thermo Fisher Scientific, USA) with an energy-dispersive X-ray spectroscopy (EDX) system for elemental analysis. Cross-sectional elemental and crystallographic analyses of the exsolved NPs were conducted using a 200 kV Jeol JEM 2100 (JEOL Ltd., Japan) transmission electron microscope equipped with a slow-scan CCD camera, a Thermo EDX detector, and a high-resolution Jeol JEM 2100F TEM.

The catalytic activity of the SFNM toward ADR was studied in a quartz tube reactor, and the resultant exhaust gases were analyzed using a quadrupole mass spectrometer (Pfeiffer Vacuum, Germany). The experimental procedure involved subjecting 0.8 g of the catalyst to a reduction in an H_2_ atmosphere at 800 °C for 20 h. Subsequently, the flow rate of 20% NH_3_ in Ar (diluting gas) was maintained at 80 mL min^–1^ STP, and the NH_3_ conversion rate was assessed over the temperature range of 550–800 °C.

### Electrochemical Characterization and Cell Performance with H_2_ and NH_3_

The electrochemical activities of the DIW 3D-printed SFNM-GDC electrodes with H_2_ and NH_3_ fuels were assessed by symmetrical half-cell testing. AC electrochemical impedance analysis (EIS) was performed using an IviumStat.XRe (Ivium Technologies, The Netherlands) instrument over the 10 mHz to 1 MHz frequency range by applying an amplitude perturbation of V_rms_ (root-mean-square) 50 mV. EIS plots for the symmetrical half-cells were analyzed at OCV across the temperature range of 600–800 °C, with a constant fuel inflow at a rate of 0.2 L min^–1^.

The EC-Lab (V11.10) fitting program analyzed the recorded data and equivalent circuits. Before performing EIS, a 1-h interval was allocated to achieve a steady-state condition, ensuring a uniform temperature distribution within the ceramic cell holder and consistent gas composition. To evaluate the performance of the symmetrical DA-SOFCs, an electrolyte-supported cell with an active electrode area of 16 cm^2^ was fabricated and analyzed. The fuel cell current–voltage-power density (*j*–*V*–*P*) and long-term stability during the galvanostatic operation were evaluated using an 850 fuel cell test station (Scribner Associates, Inc., USA).

## Supporting Information Available

XPS wide spectra of SFNM pellets after air calcination, reduction by H_2_, and eventually exposure to NH_3_ (Figure S1); O 1s and N 1s XPS spectra of SFNM pellets after air calcination, reduction by H_2_, and eventually exposure to NH_3_ (Figure S2); Resistance values obtained by modeling impedance diagram (Table S1); Arrhenius plots for each interfacial resistance (*R*_i_) of the anode reaction (Figure S3); *j*–*V*–*P* characterization of symmetrical fuel cell with undoped SFM-GDC electrodes (Figure S4); Schematics of the complete fabrication process (Figure S5) (PDF)
