# Supplementary material for: Ni-Doped SFM Double-Perovskite Electrocatalyst for High-Performance Symmetrical Direct-Ammonia-Fed Solid Oxide Fuel Cells
Source: ACS Appl Mater Interfaces. 2024 Sep 26;16(40):53652–64. doi: 10.1021/acsami.4c07968 (PMC11472266; doi:10.1021/acsami.4c07968)
Supplement: Supplementary file 1 — am4c07968_si_001.pdf [file am4c07968_si_001.pdf]

Supporting information

Ni-doped SFM double-perovskite electrocatalyst for high-performance symmetrical direct-ammonia-fed solid oxide fuel cells

*Or Rahumi[a], Manasa Kumar Rath[b], Louisa Meshi[c], Ilia Rozenblium[a], Konstantin Borodianskiy[a, *]*

[a] Department of Chemical Engineering, Ariel University, Ariel 40700, Israel

[b] Elcogen AS, 23 Valukoja, 11415, Tallinn, Estonia

[c] Department of Materials Engineering, Ben-Gurion University of the Negev, Beer-Sheva 84105, Israel

Corresponding Author

* Konstantin Borodianskiy: konstantinb@ariel.ac.il

Number of pages: 6

Number of figures: 5

Number of tables: 1

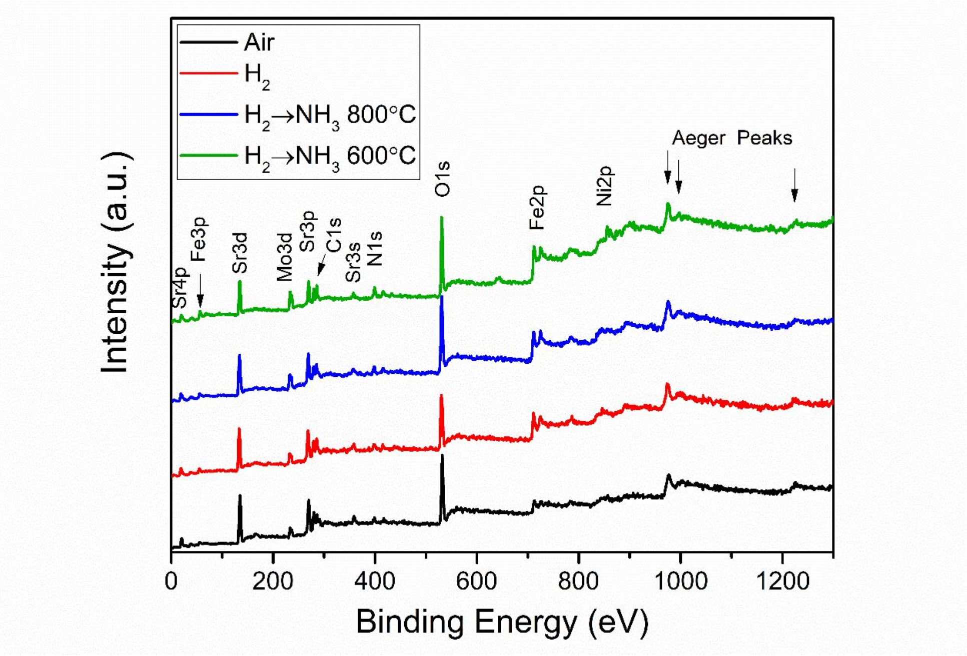

Figure S1. X-ray photoelectron spectroscopy wide spectra of $Sr_{1.9}Fe_{1.4}Ni_{0.1}Mo_{0.5}O_6$ (SFNM) pellets after air calcination, reduction by H_2, and eventually exposure to NH_3 at 800 °C and 600 °C.

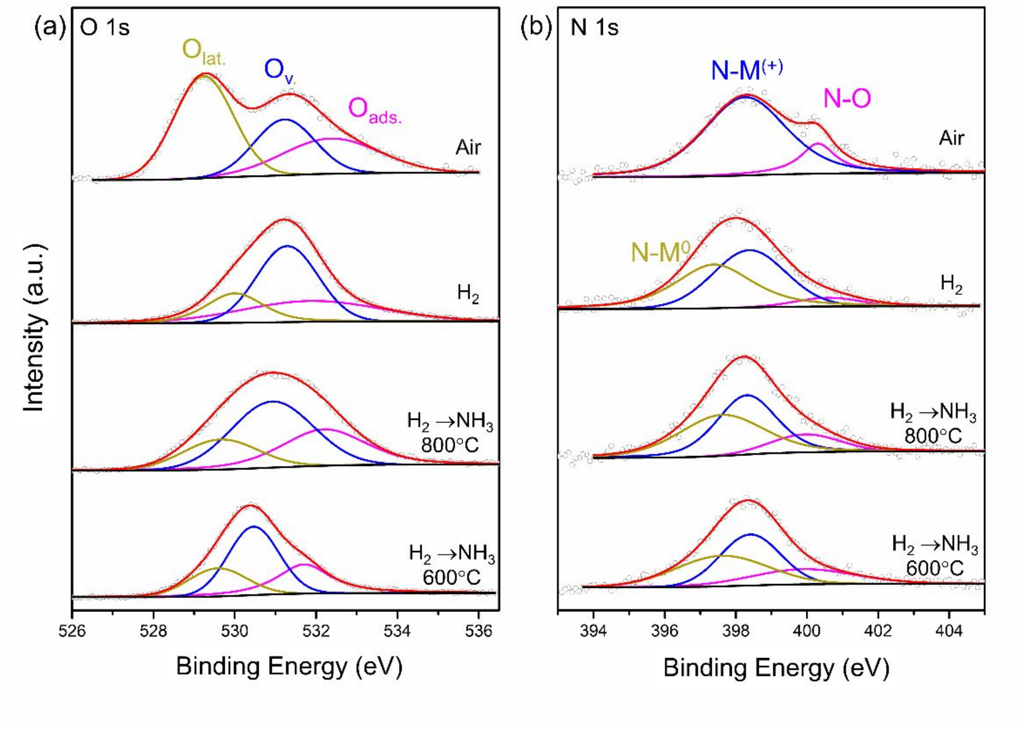

Figure S2. X-ray photoelectron spectroscopy (a) O1s and (b) N1s spectra of $Sr_{1.9}Fe_{1.4}Ni_{0.1}Mo_{0.5}O_6$ (SFNM) pellets after air calcination, reduction by H_2, and eventually exposure to NH_3 at 800 °C and 600 °C.

Table S1. Resistance values obtained by modeling impedance diagram

**Fuel**	**Temp.** [℃]	**R_{ohm}** [$\Omega \cdot cm^2$]	**R_1** [$\Omega \cdot cm^2$]	**R_2** [$\Omega \cdot cm^2$]	**Rp** [$\Omega \cdot cm^2$]	**R_2/Rp** [%]
H_2	600	8.28	1.10	6.42	7.52	85.4
	650	6.92	0.52	3.96	4.48	88.4
	700	6.00	0.23	2.35	2.58	91.1
	750	5.65	0.11	1.61	1.72	93.6
	800	5.41	0.04	1.01	1.05	96.2
NH_3	600	8.30	1.12	8.25	9.37	88.0
	650	6.77	0.50	4.20	4.70	89.4
	700	6.10	0.27	2.53	2.80	90.4
	750	5.89	0.16	1.66	1.82	91.2
	800	5.68	0.05	1.21	1.26	96.2

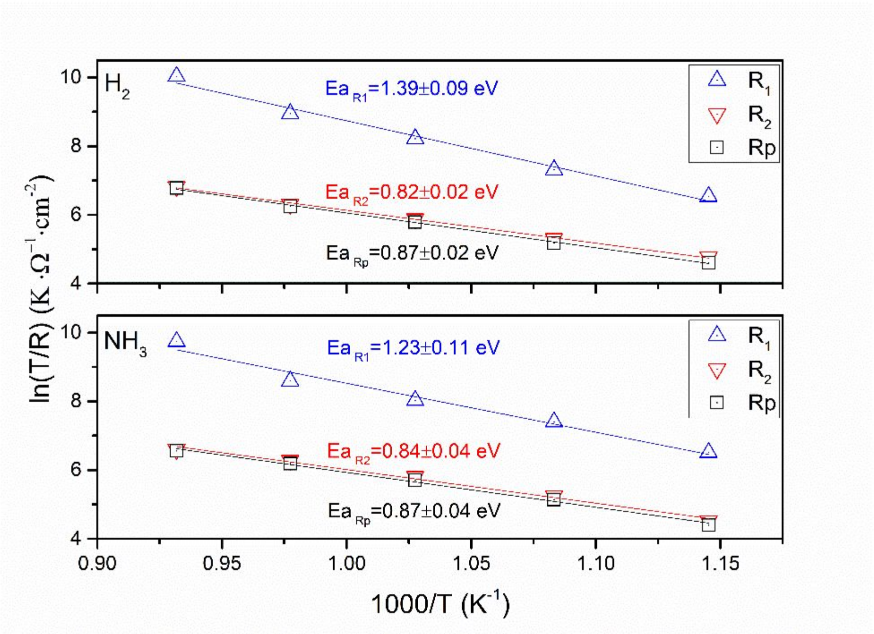

Figure S3. Arrhenius plots for each interfacial resistance (Ri) of the anode reaction fed with H_2 and NH_3 fuels.

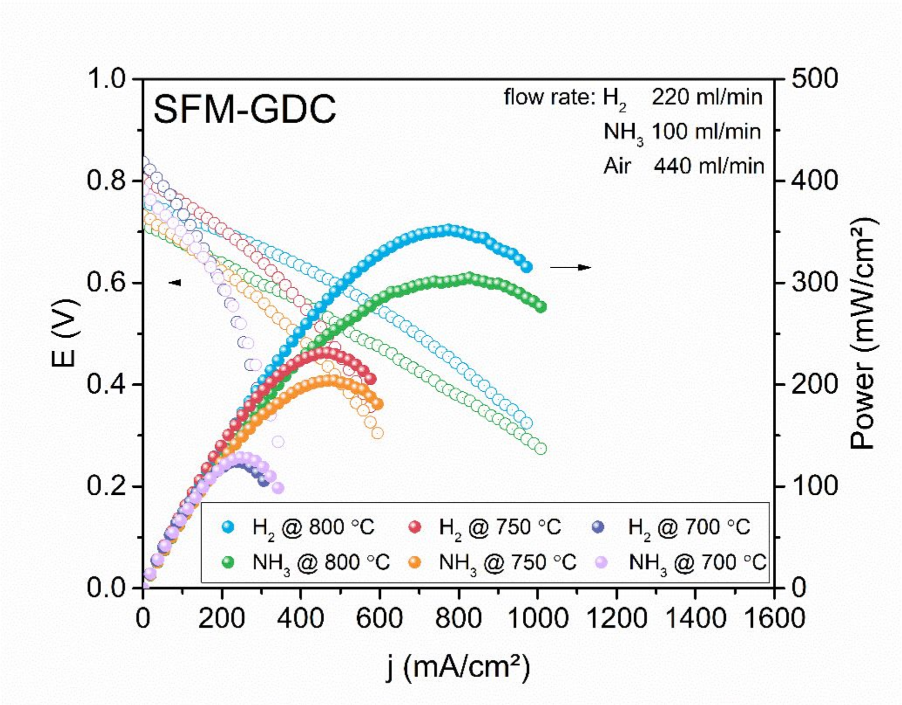

Figure S4. j-V-P characterization of symmetrical fuel cell with 200 µm GDC electrolyte and SFM ($Sr_{1.9}Fe_{1.5}Mo_{0.5}O_6$)-GDC electrodes.

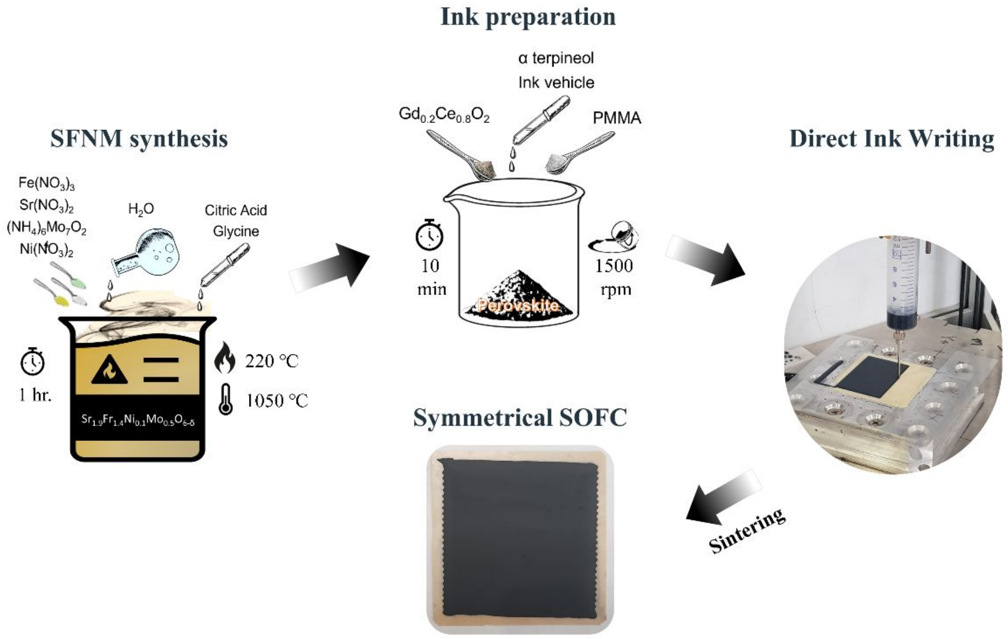

Figure S5. Schematics of the complete fabrication process includes preparation of SFNM powder, ink production, 3D printing of electrodes through DIW, and the assembly of a symmetrical cell.
